# The Role of Nrf2 and Inflammation on the Dissimilar Cardiotoxicity of Doxorubicin in Two-Time Points: a Cardio-Oncology *In Vivo* Study Through Time

**DOI:** 10.1007/s10753-023-01908-0

**Published:** 2023-10-14

**Authors:** Ana Reis-Mendes, Mariana Ferreira, Ana Isabel Padrão, José Alberto Duarte, Margarida Duarte-Araújo, Fernando Remião, Félix Carvalho, Emília Sousa, Maria Lourdes Bastos, Vera Marisa Costa

**Affiliations:** 1 Associate Laboratory i4HB - Institute for Health and Bioeconomy, Laboratory of Toxicology, Department of Biological Sciences, Faculty of Pharmacy, University of Porto, Porto, Portugal; 2 UCIBIO - Applied Molecular Biosciences Unit, REQUIMTE, Laboratory of Toxicology, Department of Biological Sciences, Faculty of Pharmacy, University of Porto, Rua de Jorge Viterbo Ferreira, 228, 4050-313 Porto, Portugal; 3 Research Center in Physical Activity, Faculty of Sport, University of Porto, Porto, Portugal; 4 Laboratory for Integrative and Translational Research in Population Health (ITR), University of Porto, Porto, Portugal; 5 1H-TOXRUN–Toxicology Research Unit, University Institute of Health Sciences, CESPU, CRL, Gandra, Portugal; 6 LAQV/REQUIMTE, University of Porto, Porto, Portugal; 7 Department of Immuno-Physiology and Pharmacology, Institute of Biomedical Sciences Abel Salazar, University of Porto, Porto, Portugal; 8 Laboratory of Organic and Pharmaceutical Chemistry, Chemistry Department, Faculty of Pharmacy, University of Porto, Porto, Portugal; 9 CIIMAR–Interdisciplinary Centre of Marine and Environmental Research, Porto, Portugal

**Keywords:** doxorubicin, inflammation, cardiotoxicity, redox homeostasis disruption, long-term effects, Nrf2.

## Abstract

Doxorubicin (DOX) is a topoisomerase II inhibitor used in cancer therapy. Despite its efficacy, DOX causes serious adverse effects, such as short- and long-term cardiotoxicity. This work aimed to assess the short- and long-term cardiotoxicity of DOX and the role of inflammation and antioxidant defenses on that cardiotoxicity in a mice model. Adult CD-1 male mice received a cumulative dose of 9.0 mg/kg of DOX (2 biweekly intraperitoneal injections (ip), for 3 weeks). One week (1W) or 5 months (5M) after the last DOX administration, the heart was collected. One week after DOX, a significant increase in p62, tumor necrosis factor receptor (TNFR) 2, glutathione peroxidase 1, catalase, inducible nitric oxide synthase (iNOS) cardiac expression, and a trend towards an increase in interleukin (IL)-6, TNFR1, and B-cell lymphoma 2 associated X (Bax) expression was observed. Moreover, DOX induced a decrease on nuclear factor erythroid-2 related factor 2 (Nrf2) cardiac expression. In both 1W and 5M, DOX led to a high density of infiltrating M1 macrophages, but only the 1W-DOX group had a significantly higher number of nuclear factor κB (NF-κB) p65 immunopositive cells. As late effects (5M), an increase in Nrf2, myeloperoxidase, IL-33, tumor necrosis factor-α (TNF-α), superoxide dismutase 2 (SOD2) expression, and a trend towards increased catalase expression were observed. Moreover, B-cell lymphoma 2 (Bcl-2), cyclooxygenase-2 (COX-2), and carbonylated proteins expression decreased, and a trend towards decreased p38 mitogen-activated protein kinase (MAPK) expression were seen. Our study demonstrated that DOX induces adverse outcome pathways related to inflammation and oxidative stress, although activating different time-dependent response mechanisms.

**Graphical Abstract:**

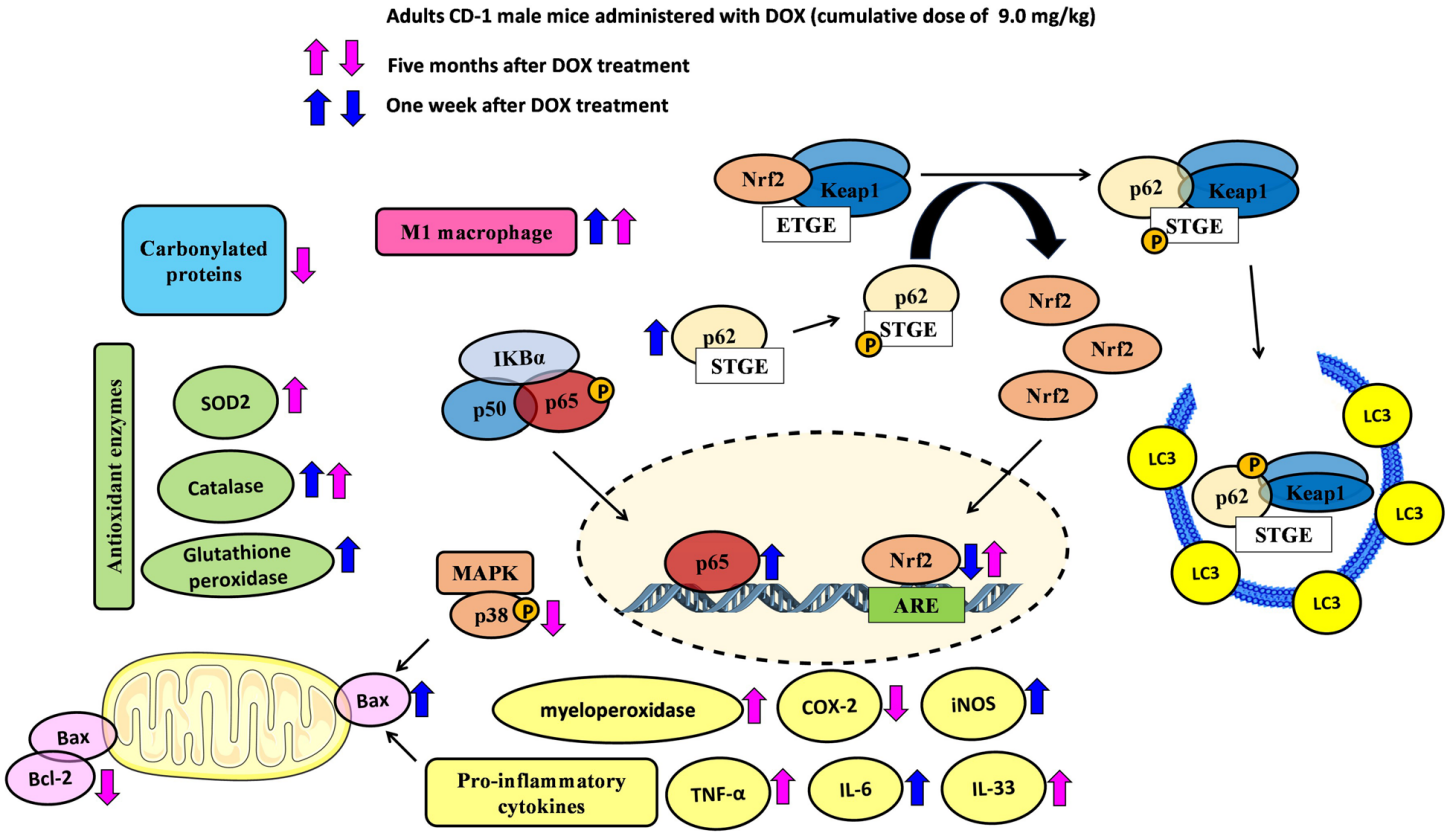

**Supplementary Information:**

The online version contains supplementary material available at 10.1007/s10753-023-01908-0.

## Introduction

Doxorubicin (DOX) is a DNA topoisomerase II inhibitor used in several types of cancers [1–3]. DOX is a powerful anticancer drug, but its use is associated with significant adverse side effects. Cardiotoxicity is one of the most critical adverse effect of DOX and the second cause of death among cancer survivors. Clinically, cardiotoxicity triggered by DOX is manifested by arrhythmias, systolic dysfunction, and heart failure (HF), among other effects [1, 2]. DOX can cause cardiotoxicity early in the treatment (up to 2 weeks after its end), being named acute/subacute cardiotoxicity. The clinical manifestations of this toxicity usually manifest themselves as chest pain, palpitation, dysplasia, and/or tachycardia arrhythmias, as well as a decline in the left ventricle ejection fraction (LVEF) [1, 2, 4]. When the effects manifest later (weeks to years after treatment has ceased), it is called chronic cardiotoxicity and is characterized by dilated cardiomyopathy, including dilation of ventricles, in some cases of atria, reduced LVEF and contractile function, diastolic dysfunction, and mural thrombi in some patients [1, 2, 4]. The incidence of late cardiotoxicity exponentially increases after the cumulative dose of 400–550 mg/m^2^ [1, 2], and DOX-induced cardiotoxicity is considered multifactorial. Several mechanisms have been suggested over the years to explain the cardiotoxicity caused by DOX: reactive oxygen species (ROS) formation has been the most reported and considered the primary mechanism of cardiotoxicity, as the chemical reactivity of DOX involves a redox cycle that results in the generation of ROS [5, 6]. On the other hand, DOX may also directly bind to endothelial nitric oxide (^•^NO) synthase and generate a DOX-semiquinone radical [7]. Mitochondrial DNA lesions caused by ROS or directly by DOX can lead to respiratory chain failure and ROS formation [8]. Nonetheless, antioxidants do not revert the inflicted damage when experimental and clinical trials address the long-term effects of the drug [9, 10]. Other contributors to DOX-induced cardiotoxicity include inhibition of nucleic acid and protein synthesis, the release of vasoactive substances, disturbed function of myocardial adrenergic receptors and adenylate cyclase activity, alterations in lysosomal morphology and enzyme activities, disruption of calcium transport in the cardiac sarcolemma, accumulation of iron in mitochondria, the formation of cardiotoxic metabolites and the activation of apoptosis [11, 12]. However, extensive research has revealed that DOX may unleash alternative cardiac damaging mechanisms, such as cardiac topoisomerase 2β inhibition, autophagy, pyroptosis, immunometabolism, and inflammation [13–16].

Moreover, the classic vision has been that DOX-induced cardiac inflammation occurs due to the production of ROS and the subsequent apoptosis of cardiomyocytes [17], which provokes an increase in pro-inflammatory cytokines, inflammatory cell infiltration, and necrosis in cardiac tissues [18]. Accordingly, in our previous *in vivo* work, which used clinically relevant doses, we showed that adult mice seem to be more prone to DOX-induced cardiotoxicity, by mechanisms related to inflammation when compared with infant mice [19]. Several *in vivo* studies using short-term administrations have demonstrated that DOX leads to inflammation of the cardiac tissue [20–22]. However, as far as we know, the role of inflammation in the long-term cardiotoxicity of DOX has yet to be determined.

Moreover, we demonstrated that DOX causes a cardiac inflammatory response and changes the hearts’ redox response [19]. Inflammation is closely linked to cardiac redox homeostasis, being that the transcription factor nuclear factor erythroid 2-related factor 2 (Nrf2) is a paramount link. Nrf2 is a regulator of multiple cytoprotective genes, which maintain redox homeostasis and exert anti-inflammatory functions; thus, it is a strong contributor to cardiovascular health [23]. Over the last few years, several studies have supported the regulation of DOX cardiotoxicity by Nrf2 [24–26]. The activation of the Keap1/Nrf2 antioxidant response system has been identified as an important cellular defense mechanism against oxidative stress after acute exposure to DOX *in vitro* [27]. Among the antioxidant enzyme expression regulated by Nrf2 are catalase, superoxide dismutase, glutathione S-transferases, and glutathione peroxidases, among other redox regulators [23, 28]. In addition, the Nrf2 signaling pathway exerts a negative regulatory influence on various inflammatory mediators such as cytokines, chemokine-releasing factors, matrix metalloproteinases, cyclooxygenase-2 (COX-2), and inducible nitric oxide synthase (iNOS). These mediators, in turn, directly or indirectly impact inflammation-controlling networks, including nuclear factor κB (NF-κB) and mitogen-activated protein kinase (MAPK) pathways, among others [28, 29]. Therefore, and considering the existing knowledge, this study aimed to evaluate the role of inflammation in DOX-induced cardiotoxicity and its possible link to its underlying late cardiotoxicity by using a clinically relevant dose in adult mice. Hence, Nrf2, NF-κB, and other redox and inflammation responders were assessed herein to gain a deeper disclose of the pathways involved in DOX-inflicted cardiotoxicity, mainly in the long-term. As far as we know, no other pre-clinical study evaluated the impact of DOX 5 months after the last administration.

## Materials and Methods

Doxorubicin hydrochloride (≥ 98% purity, DOX), Ponceau S, direct red 80, 5,5-dithiobis(2-nitrobenzoic acid), adenosine triphosphate (ATP), reduced glutathione (GSH), glutathione reductase, oxidized glutathione (GSSG) disodium salt, bovine serum albumin, and the all other chemicals used were purchased from Sigma-Aldrich (St. Louis, MO, USA). Phosphate-buffered saline solution was purchased from Biochrom (Berlin, Germany), and sodium chloride (NaCl) was acquired from VWR (Leuven, Belgium). Isoflurane (Isoflo^®^) was obtained from Abbott Animal Health (North Chicago, IL, USA). Harris haematoxylin was purchased from Harris Surgipath (Richmond, IL, USA), and 1% aqueous eosin from Australian Biostain (Traralgon, Australia). The Bio-Rad DC protein assay kit was obtained from Bio-Rad Laboratories (Hercules, CA, USA). Primary antibodies were acquired from different sources which are indicated in Supplementary Table S1. Goat anti-rabbit IgG-horseradish peroxidase (ab97051) and goat anti-mouse IgG-horseradish peroxidase (ab6728) were provided by Abcam (Cambridge, UK), while enhanced chemiluminescence (Clarity Western ECL, 1.705.060) reagents and the Bio-Rad DC protein assay kit were purchased from Bio-Rad Laboratories (Hercules, CA, USA). Amersham Protran nitrocellulose blotting membranes (0.45 µm) were supplied by Cytiva (Buckinghamshire, UK).

### Animals

Male CD-1 mice (*Mus musculus*) were acquired from Charles River Laboratories (L’Arbresle, France) and housed in the rodent animal house facility of the Institute for Biomedical Sciences Abel Salazar, University of Porto (ICBAS-UP). Animals were housed under controlled temperature (22 ± 2 °C) and humidity (55% ± 10) in a light–dark cycle of 12 h in IVC Sealsafe plus mouse Green Mouse 500 cages. Animals were given *ad libitum* access to water and standard rodent chow 4RF21 GLP certificate diet (Mucedola, Settimo Milanese, Italy). A week before the first administration of the drug or vehicle, the mice were accustomed to the environmental conditions and handling researchers to decrease stress and increase animal well-being. This study was carried out in accordance with Portuguese law (Decreto-Lei no. 113/2013, which follows the European Directive 2010/63/EU), and the project was authorized by the competent local (ORBEA ref. 140/2015) and national (DGAV, ref. 021322 of 26 October 2016) entities responsible for animal welfare.

### Study Design

Adult male mice (*n* = 30) at 12 weeks were used in this study. All animals were given six intraperitoneal (ip) injections (two *per* week) with saline solution (NaCl 0.9%, control group) or DOX to reach a total cumulative dose of 9.0 mg/kg (DOX group, DOX was solubilized in sterile NaCl 0.9%). Allometric scaling was used to ensure that the administered cumulative dose did not exceed the maximum recommended cumulative doses for human DOX therapy [30]. The mice were randomized into one of the following experimental protocols:

- Short period protocol: 6 mice were sacrificed one week after the last administration of DOX (1W-DOX) and 6 mice were used as the control group (1W-Control);
- Long period protocol: 9 mice were sacrificed five months after the last administration of DOX (5M-DOX) and 9 mice were used 5 months after the last saline administration, being the control group (5M-Control).

According to the literature, at the beginning of the protocol, the age of adult mice (12 weeks) was similar to that of young human adults (around 20 human years), as mice reach sexual maturity at an average age of 10 weeks [31]. After the last administration, mice were kept in a drug-free period until sacrifice, for a short period (1 week (1W)) or a long period (5 months (5M)). The animals sacrificed 1W were approximately 4 months old at the time of sacrifice, which corresponds approximately to 24 human years. Animals included in the long period (5M) protocol were 9 months old at the time of sacrifice, which roughly corresponds to 36 human years [32].

### Administration Schedule and Experience Follow-up

Our administration schedule was chosen to mimic a human therapy scheme (multiple administrations at separate time points), which allows us to evaluate toxicity induced by a cumulative dose over time, rather than an acute drug response. The 9.0 mg/kg cumulative dose of DOX in mice roughly corresponds to 54.45 mg/m^2^ in humans [30, 33, 34]. This dose is much lower than the maximum lifelong dose recommended for humans (400–550 mg/m^2^) [1]. We used a low, clinically relevant cumulative dose and took our study further to investigate the impact of DOX 5 months after its last administration.

Throughout the experimental protocol, water and food consumption, body weight, and the welfare of the mice were routinely evaluated by at least two researchers. Regarding animal welfare, human endpoints, a grimace scale, administration response, and an adapted scoring system that included assessment of the animals’ general activity, distress, body condition, and presence of diarrhea or ascites (described previously by this research group [33]) were applied.

At the end of the protocol, animals were deeply anesthetized with isoflurane *ad effectum* and then sacrificed by exsanguination. The heart and brain were removed and weighed. The brain was used to assess the heart weight-to-brain weight ratio. The heart was separated into several pieces and then processed for biochemical, histological, immunohistochemical, and immunoblotting analysis, as previously described by us [19, 33].

### Measurements of ATP, tGSH, GSH, and GSSG Levels

ATP levels were evaluated by a bioluminescent assay based on the luciferin-luciferase reaction, and total glutathione (tGSH) and GSSG levels were evaluated by the 5,5-dithiobis(2-nitrobenzoic acid)-GSSG reductase recycling assay, as previously described [33, 35]. GSH levels were calculated using the formula: GSH = tGSH – 2 × GSSG. The results of ATP, tGSH, GSH, and GSSG were normalized to the total protein content and expressed as nmol of ATP or tGSH or GSSG *per* mg of protein (nmol ATP/mg protein or nmol GSH/mg protein or nmol GSSG/mg protein). Protein content in the homogenate was quantified using the Lowry method [36], and bovine serum albumin as the standard.

### Histological Analysis

All histological procedures were conducted according to previously published procedures [19, 33, 35]. Serial cardiac cross-sections of paraffin blocks were subjected to two types of staining: haematoxylin and eosin for routine histological evaluation and Sirius red for collagen tissue staining. The slides were examined and photographed with a Carl Zeiss Imager A1 light microscope equipped with an AxioCam MRc 5 digital camera (Oberkochen, Germany). Sections stained with haematoxylin and eosin were used to evaluate cardiac tissue damage. Histopathological evidence of tissue damage was calculated according to severity and incidence in every slide, as previously published [19, 33, 35]. To semi-quantify the severity of the damage in cardiac tissue, the slides were analyzed in a blinded fashion regarding the following parameters: (i) cellular degeneration, (ii) interstitial inflammatory cell infiltration, (iii) necrotic zones, and (iv) tissue organization, as previously published [19, 33, 35]. The severity of cellular degeneration was scored according to the total of cells that showed changes (dilatation, vacuolization, pyknotic nuclei, and cellular density) in the microscopy visual field, using a score from 0 to 3. Tissue necrosis severity, tissue disorganization, and inflammatory activity were also scored (from 0 to 3 values) according to the quantity of tissue affected [19, 33, 35].

Sections stained with Sirius Red were used to assess collagen deposition. The images were evaluated using ImageJ software (version 1.52a, Wayne Rasband, NIH, Bethesda, Maryland, USA) and the results of collagen seen as red staining are expressed as a percentage of collagen *per* total section area, as previously detailed [19, 33].

### Immunohistochemistry Analysis

The detection of M1 and M2 macrophages and NF-κB p65 in the heart tissue was conducted by immunohistochemistry, as previously published [33]. The slides were analyzed in a Carl Zeiss Imager A1 light microscope and images were recorded with a coupled AxioCam MRc 5 digital camera (Oberkochen, Germany).

### Immunoblotting Analysis

Western blotting and slot blot analysis were performed according to what has been previously published [19, 33]. Treated animals and respective controls were analyzed on the same membrane. Immunoreactive bands were detected by using the enhanced chemiluminescence ECL reagents, according to the manufacturer’s instructions. The immunoreactive bands were automatically detected using the ChemiDoc Imaging System version 2.3.0.07 (Bio-Rad, Hercules, CA, USA). The images obtained were analyzed using the Image Lab software version 6.0.1 (Bio-Rad, Hercules, CA, USA). Protein content in the homogenate was quantified using the Bio-Rad DC Protein assay. Protein loading was confirmed by Ponceau S staining.

### Statistical Analysis

Results were expressed as mean ± standard deviation (SD). Statistical analyses of the animal body weight, food, and water intake data were carried out by the two-way analysis of variance (2-way ANOVA) followed by the Sidak *post hoc* test. To assess data normality on assays, the Shapiro–Wilk test normality test was performed. When two groups were analyzed, the unpaired *t*-test was used when the distribution was normal or by the Mann–Whitney test when the distribution was not normal. The outliers were identified using the ROUT method (*Q* = 1%), and then, statistical analysis was performed. Statistical significance was considered with *p* values < 0.05. For *p* values < 0.1, a tendency was assumed. To perform the statistical analysis, GraphPad Prism software (version 8.4.2) (San Diego, CA, USA) was used.

## Results

### Mice Body Weight Was Affected 5M After the Last Administration of DOX

**Fig. 1:**
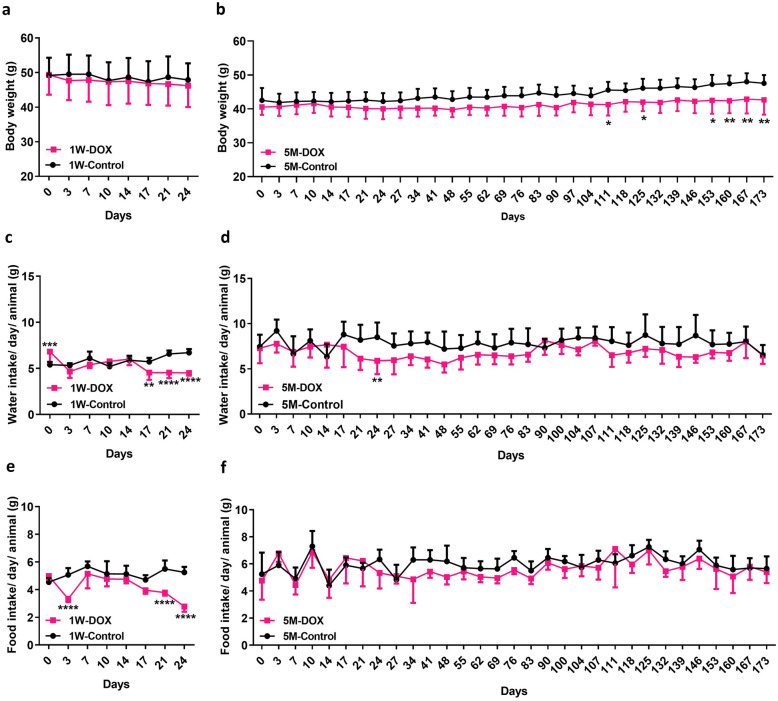
Body weight (**a**, **b**), water (**c**, **d**), and food (**e**, **f**) intake of mice sacrificed 1 week after the last administration of DOX (1W-DOX) and control mice (1W-Control); and mice sacrificed 5 months after the last administration of DOX (5M-DOX) and respective controls (5M-Control). DOX is shown in pink squares while controls are shown in black circles. Results are presented in grams (g) of body weight, mL of water intake/day/weight/animal, and g of food intake/day/weight/animal, as means ± SD. Statistical comparisons were made using a two-way ANOVA followed by Sidak’s *post hoc* test (*p < 0.05, **p < 0.01, ***p < 0.001, and ****p < 0.0001, DOX *vs*. control).

In the animals sacrificed 5M after the last administration, there was a significant body weight decrease in the DOX-treated animals compared to controls in the last days of the protocol (Fig. 1b). Some animals in this group presented piloerection, weakness, and a hunched posture. When human endpoints were reached (according to the scoring system previously described [33]), the animals were humanely sacrificed with an isoflurane overdose. In the protocol of the 5M animals, one mouse died suddenly (41 days after starting the protocol) and was not considered for data analysis, but post-mortem examination showed pleural effusions and dilated myocardium. Regarding the body weight of the 1W-DOX group, it was constant throughout the experiment, that is, DOX and control groups did not show significant differences in total body weight (Fig. 1a).

All water and food consumptions were normalized relative to body weight, assuming that all animals had intake rates proportional to their current weights. The 1W-DOX group showed significantly lower water consumption from the 17th day until the end of the experiment (Fig. 1c), compared to controls. In the 5M-DOX group, water intake tended to be lower in the first weeks of the protocol (Fig. 1d).

The 1W-DOX group had lower consumption of food from day 21 onwards, compared to controls (Fig. 1e). Regarding the 5M groups, the food consumption was constant through the protocol, that is, DOX and control groups did not show significant differences in food consumption (Fig. 1f).

Finally, mice that were included in both 1W-DOX and 5M-DOX groups showed a heart weight-to-brain weight ratio that did not differ from controls (data not shown).

### The 1W-DOX Group Had Significant Histological Changes

**Fig. 2:**
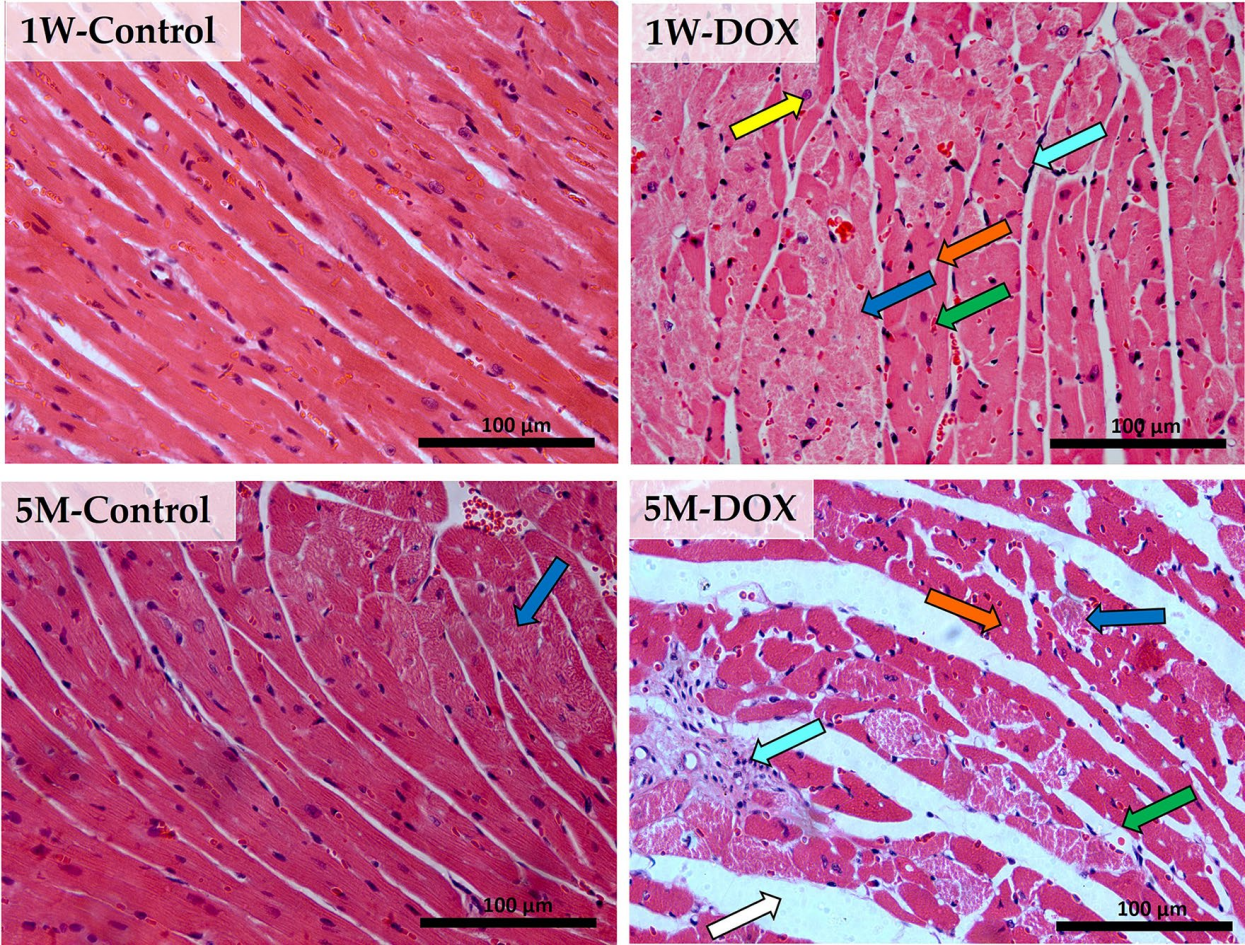
Representative light microscope micrographs were obtained after haematoxylin and eosin staining of the heart of mice, sacrificed 1 week after the last administration of DOX (1W-DOX) and control mice (1W-Control); and mice sacrificed 5 months after the last administration of DOX (5M-DOX) and respective controls (5M-Control), assessed by the haematoxylin and eosin staining. Control mice at 1W showed normal morphology and structure; mice given a cumulative dose of 9.0 mg/kg cumulative dose of DOX presented large and uncondensed nuclei (yellow arrow), interstitial oedema (white arrow), vacuolization (orange arrow), inflammatory infiltration (cyan arrow), and vascular congestion (green arrow). Necrotic zones (blue arrow) are evident. Scale bar = 100 µm, *n* = 3. Images were taken at 40 × magnification.

Histologic examination by light microscopy of the cardiac morphology (Fig. 2) showed that control hearts (1W-Control) were normal: the myocardial cells in the control group were arranged neatly, with no bleeding, oedema, and other abnormalities. However, at 5M, the contol group of hearts showed some necrotic zones. Nonetheless, in DOX groups, alterations in the cardiac tissue were microscopically characterized by cellular degeneration, interstitial inflammatory cell infiltration, necrotic zones, and loss of tissue organization. In 1W-DOX and 5M-DOX groups, swollen and vacuolated cardiomyocytes, intracellular oedema, and disorganization of myofibrils, as well as some necrotic zones were seen. Nevertheless, the 1W-DOX group showed significant lesions compared to their control; at 5M, the DOX animals did not show significant damage in several parameters of the semi-quantitative analysis (Table 1) but damage is clearly seen on the light micrographs (Fig. 2).

**Table 1:** Semi-quantitative Analysis of the Morphological Parameters of the Cardiac Tissue of Mice of Both Protocols

Haematoxylin–eosin staining	1W-Control	1W-DOX	5M-Control	5M-DOX
Cellular degeneration	0.02	0.47****	0.08	0.13
Necrotic zones	0.00	0.42****	0.13	0.37*
Infiltration of interstitial inflammatory cells	0.05	0.46****	0.10	0.20
Loss of tissue organization	0.00	0.14 (*p* = 0.054)	0.03	0.10

Semi-quantitative analysis of several morphological parameters in the cardiac tissue of mice sacrificed 1 week after the last administration of DOX (1W-DOX) and control mice (1W-Control), and mice sacrificed 5 months after the last administration of DOX (5M-DOX) and respective controls (5M-Control). Slides were analyzed for the following parameters: (i) cellular degeneration, (ii) necrotic zones, (iii) infiltration of interstitial inflammatory cells, and (iv) tissue organization, using a score of 0 to 3. Results are presented as means of scores (*n* = 3 animals *per* group, ten random fields *per* animal). Statistical comparisons were made using the Mann–Whitney test: *p < 0.05, ****p < 0.0001, DOX *vs.* control at the same time-point

### DOX Treatment Induced Myocardial Fibrosis in 1W and 5M

**Fig. 3:**
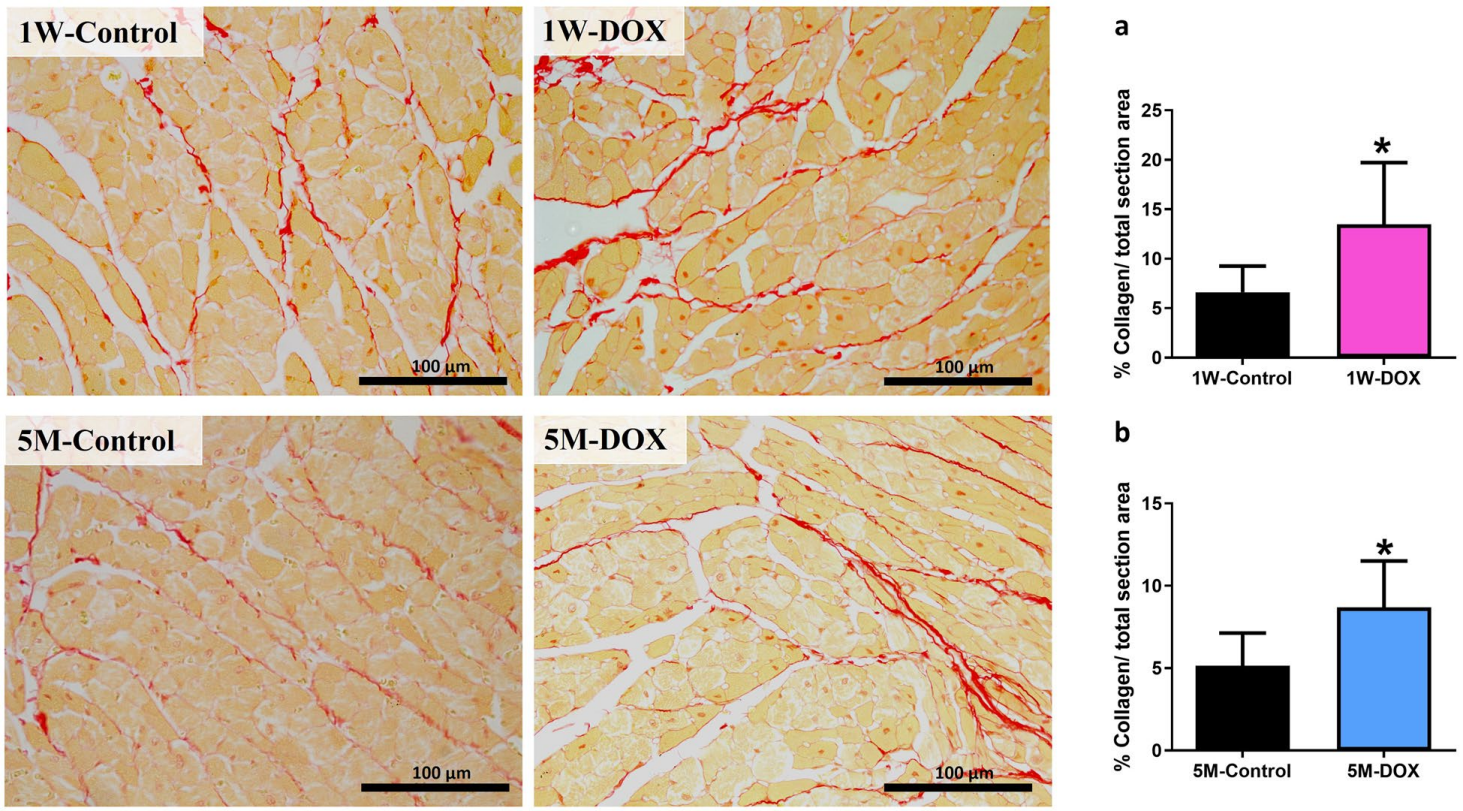
Representative light microscope micrographs were obtained after Sirius red staining of the heart of mice sacrificed one week after the last administration of DOX (1W-DOX) and control mice (1W-Control), and mice sacrificed 5 months after the last administration of DOX (5M-DOX) and respective controls (5M-Control). Scale bar = 100 µm, *n* = 3 animals *per* group, six random fields *per* animal. Images were taken at 40 × magnification. **a**–**b** The results of collagen content red staining are expressed as a percentage of collagen *per* total section area and are presented as means ± SD. Statistical comparisons were made using the Mann–Whitney test: *p < 0.05, DOX *vs*. control.

As shown in Fig. 3, 1W-DOX and 5M-DOX groups showed a significant increase in the percentage of collagen *per* total section area compared with the controls (1W-Control and 5M-Control).

### No Significant Changes in the Cardiac Levels of ATP or Glutathione-Related Parameters Were Observed in 1W-DOX and 5M-DOX Groups

As shown in Table 2, no significant differences were found in the cardiac levels of ATP, tGSH, and GSSG in the DOX-treated mice of both groups. Nonetheless, the 5M-DOX group showed a significant increase in the GSH/GSSG ratio compared with the controls (5M-Control).

**Table 2:** Biochemical Cardiac Parameters of the DOX-Treated and Control Mice

	**1W-Control**	**1W-DOX**	**5M-Control**	**5M-DOX**
tGSH (nmol/ mg protein)	4.207 ± 2.277	3.527 ± 2.301	3.343 ± 1.615	3.358 ± 1.193
GSSG (nmol/ mg protein)	0.578 ± 0.263	0.573 ± 0.299	3.314 ± 1.552	2.403 ± 0.592
GSH/ GSSG ratio	7.257 ± 1.330	6.338 ± 3.227	1.020 ± 0.331	1.572 ± 0.336*
ATP (nmol/ mg protein)	0.280 ± 0.183	0.189 ± 0.068	0.237 ± 0.063	0.247 ± 0.045

Biochemical cardiac parameters of mice sacrificed 1 week after the last administration of DOX (1W-DOX) and control mice (1W-Control), and mice sacrificed 5 months after the last administration of DOX (5M-DOX) and respective controls (5M-Control). Results, in ratio or nmol/mg protein, are presented as means ± SD and were obtained from 6 (1W) to 7–9 (5M) animals from each treatment group. Statistical analyses were made using the unpaired *t*-test (5M): *p < 0.05, DOX *vs*. control at the same time-point

### The 1W-DOX Group Showed a Significant Increase in Glutathione Peroxidase, Catalase, and iNOS Expression, While the 5M-DOX Group Showed a Significant Increase in Superoxide Dismutase Expression

**Fig. 4:**
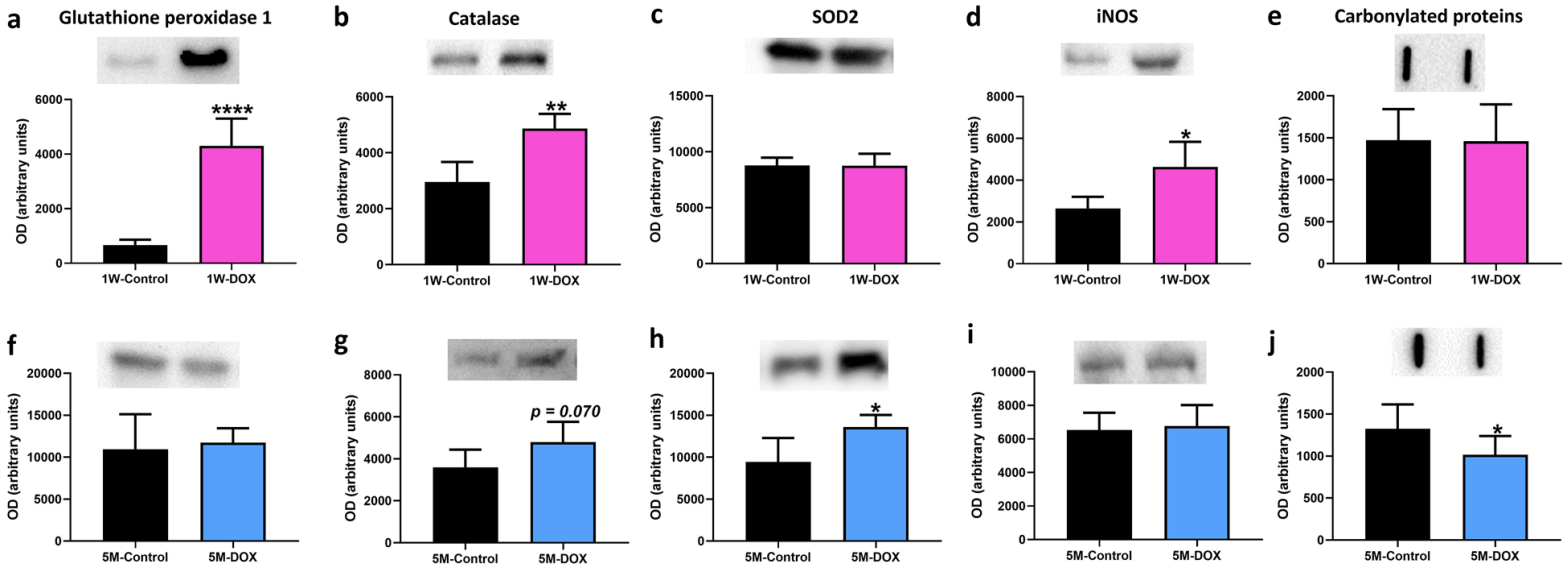
Western blotting analysis of **a**, **f** glutathione peroxidase (22 kDa), **b**, **g** catalase (60 kDa), **c**, **h** superoxide dismutase 2 (SOD2) (26.6 kDa) and **d**, **i** inducible nitric oxide synthase (iNOS) (131 kDa) expression in the cardiac tissue. **e**, **j** Protein carbonylation cardiac content was evaluated by slot blot. **a**, **b**, **c**, **d**, **e** Mice sacrificed 1 week after the last administration of DOX (1W-DOX) and control mice (1W-Control); and **f**, **g**, **h**, **i**, **j** mice sacrificed 5 months after the last administration of DOX (5M-DOX) and respective controls (5M-Control). Values are expressed as mean ± SD and were obtained from 4–6 (1W) to 4–7 (5M) animals from each treatment group. Statistical comparisons were made using the unpaired *t*-test: *p < 0.05, **p < 0.01, ****p < 0.0001, DOX *vs*. control. OD, optic density. Protein loading was confirmed by the Ponceau S staining (Fig. S1).

As shown in Fig. 4, glutathione peroxidase, catalase, and iNOS expression were significantly increased in the 1W-DOX group when compared with 1W-Control (Fig. 4a, b, d). The 1W-DOX group had no significant changes in SOD2 expression (Fig. 4c). The 5M-DOX group showed no meaningful differences in glutathione peroxidase and iNOS expression (Fig. 4f, i). The 5M-DOX group showed a significant increase in SOD2 expression (Fig. 4h) and a tendency for increased (*p* = 0.070) catalase expression in comparison with the control group (Fig. 4g).

### Protein Carbonylation in Cardiac Lysates Decreased Significantly in the 5M-DOX Group

The 1W-DOX group showed no meaningful differences in carbonylated protein expression compared to the 1W-Control mice (Fig. 4e). The protein carbonylation in the cardiac lysates decreased significantly in the 5M-DOX group, compared to 5M-Control mice (Fig. 4j).

### The 1W-DOX Group Showed a Significant Decrease in Nrf2 Expression, While It Increased Significantly in the 5M-DOX Group

**Fig. 5:**
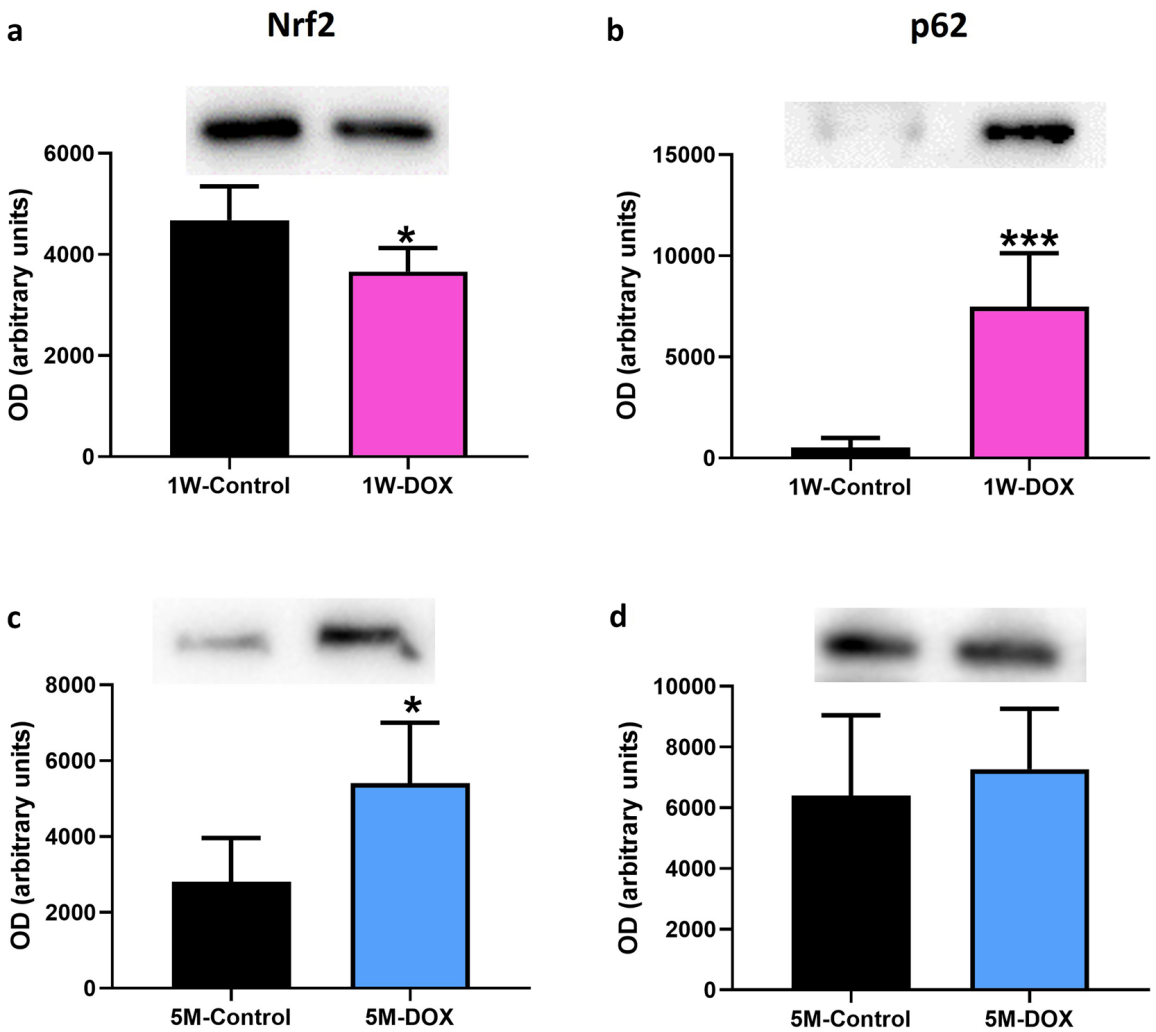
Western blotting analysis of **a**, **c** nuclear factor erythroid-2 related factor 2 (Nrf2) (97 kDa) and **c**, **d** p62 (62 kDa) expression in the cardiac tissue, in **a**, **b** mice sacrificed 1 week after the last administration of DOX (1W-DOX) and control mice (1W-Control); and **c**, **d** mice sacrificed 5 months after the last administration of DOX (5M-DOX) and respective controls (5M-Control). Values are expressed as mean ± SD and were obtained from 4–6 (1W) to 5–6 (5M) animals from each treatment group. Statistical comparisons were made using the unpaired *t*-test: *p < 0.05, ***p < 0.001, DOX *vs*. control. OD, optic density. Protein loading was confirmed by the Ponceau S staining (Fig. S2).

In the 1W-DOX group, Nrf2 expression decreased significantly, and p62 expression increased significantly when compared with 1W-Control mice (Fig. 5a, b). In the 5M-DOX group, Nrf2 expression increased significantly when compared with the 5M-Control mice (Fig. 5c), although no significant differences (Fig. 5d) in p62 expression were seen.

### Both 1W-DOX and 5M-DOX Groups Showed a Higher Density of Infiltrating M1 Macrophages in Cardiac Tissue

**Fig. 6:**
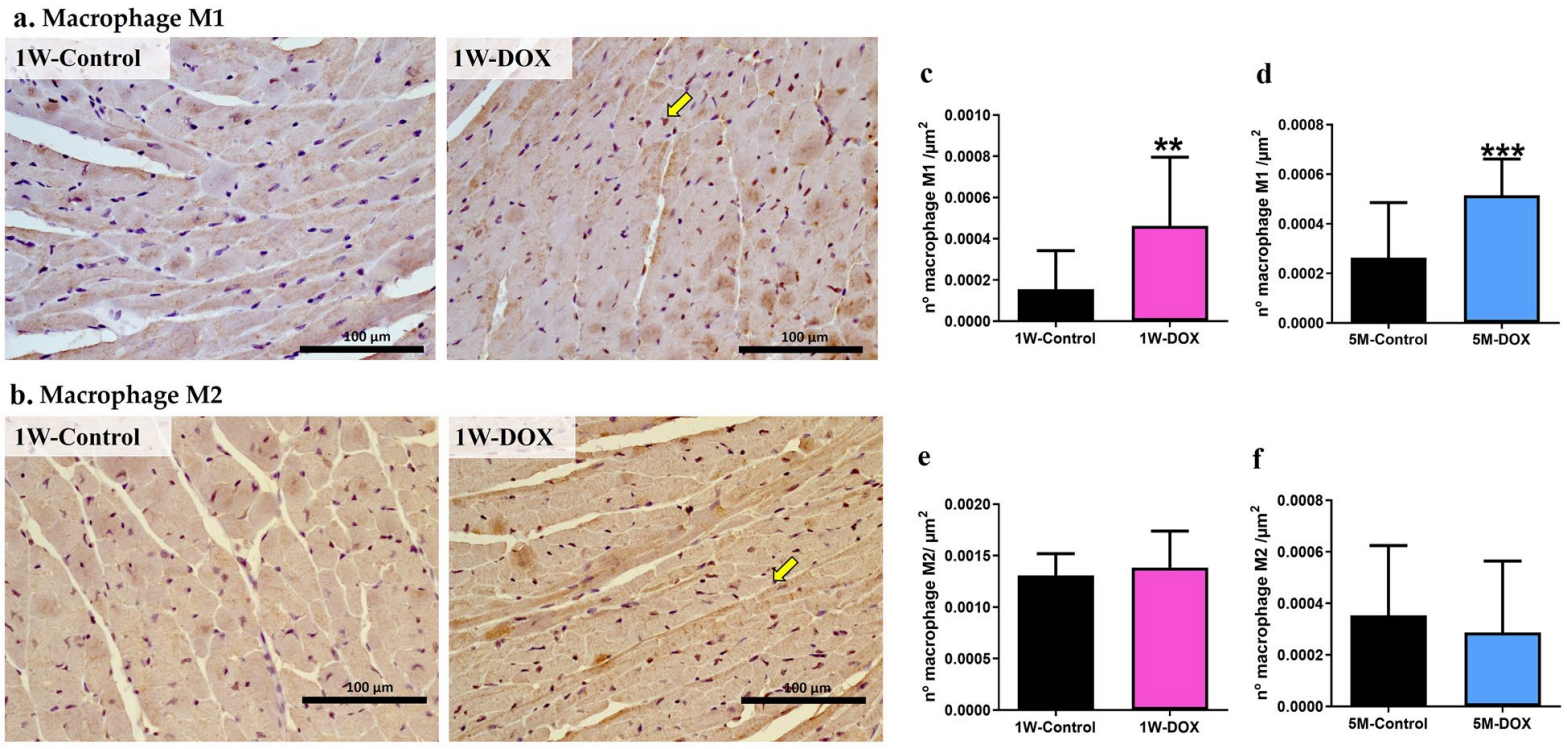
**a** Representative images of the immunohistochemistry determination of macrophages markers CD68 (a marker for the macrophage M1) (on the top line) and **b** CD206 (a marker for the macrophage M2) (the bottom histologic figures) in cardiac tissue of mice sacrificed 1 week after the last administration of DOX (1W-DOX) and control mice (1W-Control). **c**, **d** The number of cells staining as positive, indicated by yellow arrows for the activated macrophages marked as M1 of the heart of DOX-treated and control groups, **c** 1W-DOX and 1W-Control; and **d** 5M-DOX and respective 5M-Control. **e**, **f** The number of cells staining as positive, indicated by yellow arrows for the activated macrophages marked as M2 of the heart of DOX-treated and control groups, **e** 1W-DOX and 1W-Control; and **f** 5M-DOX and respective 5M-Control. The results were expressed according to the number of positive cells *per* area (µm^2^) as mean ± SD. Statistical comparisons were made using the Mann–Whitney test: **p < 0.01, ***p < 0.001, DOX *vs.* control. Scale bar = 100 µm, *n* = 3 animals *per* group, six random fields *per* animal. Images were taken at 40× magnification.

The number of CD68-positive cells increased significantly in both groups treated with DOX (Fig. 6a, b) when compared to the respective controls. No meaningful differences were observed in the number of M2 macrophages in groups (Fig. 6c, d) treated with DOX when compared to the respective controls.

### The 1W-DOX Group Showed a Significant Increase in TNFR2 Expression, While 5M-DOX Mice Had Increased IL-33 and TNF-α Expression

**Fig. 7:**
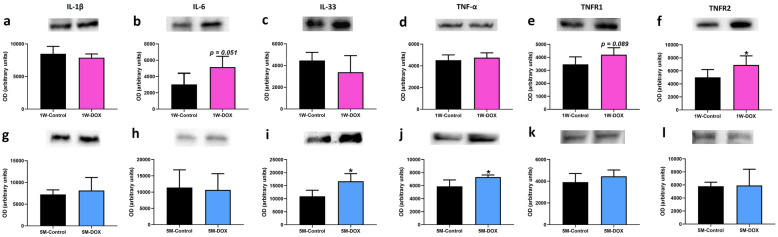
Western blotting analysis of **a**, **g** interleukin-1β (IL-1 β) (35 kDa), **b**, **h** interleukin-6 (IL-6) (23 kDa), **c**, **i** interleukin-33 (IL-33) (33 kDa), **d**, **j** tumor necrosis factor-α (TNF-α) (25 kDa), **e**, **k** type 1 TNF receptor (TNFR1) (50 kDa) and **f**, **l** type 2 TNF receptor (TNFR2) (75 kDa) expression in the cardiac tissue, in **a**, **b**, **c** mice sacrificed 1 week after the last administration of DOX (1W-DOX) and control mice (1W-Control); and **d**, **e**, **f** mice sacrificed 5 months after the last administration of DOX (5M-DOX) and respective controls (5M-Control). Values are expressed as mean ± SD and were obtained from 4–6 (1W) to 4–6 (5M) animals from each treatment group. Statistical comparisons were made using the unpaired *t*-test: *p < 0.05, DOX *vs*. control. OD, optic density. Protein loading was confirmed by the Ponceau S staining (Fig. S3).

In the 1W-DOX group, no changes in interleukin (IL)-1β, IL-33, and tumor necrosis factor-α (TNF-α) expression (Fig. 7a, c, d) were seen when compared to 1W-Control mice. On the other hand, the 1W-DOX group tended to increase IL-6 expression (*p* = 0.051) (Fig. 7b) and type 1 TNF receptor (TNFR1) expression (*p* = 0.089) (Fig. 7e) when compared to 1W-Control mice. Moreover, type 2 TNF receptor (TNFR2) expression increased significantly in 1W-DOX mice, when compared with the 1W-Control mice (Fig. 7f). Furthermore, the 5M-DOX group had significantly increased IL-33 and TNF-α expression (Fig. 7i, j) compared to 5M-Control mice. No meaningful differences in IL-1β, IL-6, TNFR1, and TNFR2 expression were seen in that latter time point (Fig. 7g, h, k, l).

### The 5M-DOX Group Had a Significant Decrease in COX-2 Expression, While Myeloperoxidase Expression Increased Significantly

**Fig. 8:**
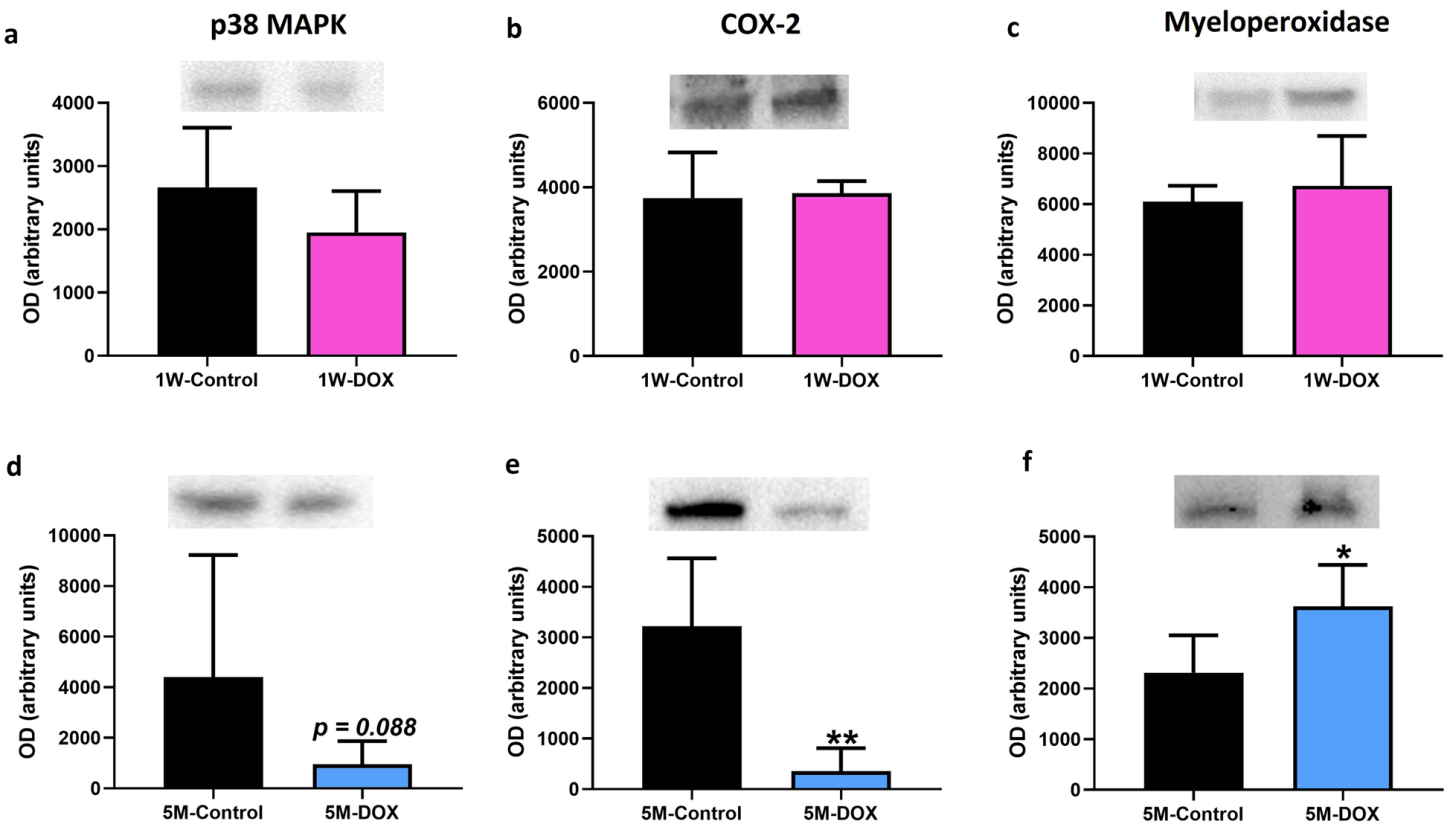
Western blotting analysis of **a**, **d** p38 mitogen-activated protein kinase (p38 MAPK) (40 kDa), **b**, **e** cyclooxygenase-2 (COX-2) (75 kDa), **c**, **f** myeloperoxidase (63 kDa) expression in the cardiac tissue, in **a**, **b**, **c** mice sacrificed 1 week after the last administration of DOX (1W-DOX) and control mice (1W-Control); and **d**, **e**, **f** mice sacrificed 5 months after the last administration of DOX (5M-DOX) and respective controls (5M-Control). Values are expressed as mean ± SD and were obtained from 4–6 (1W) to 5–7 (5M) animals from each treatment group. Statistical comparisons were made using the unpaired *t*-test: *p < 0.05, **p < 0.01, DOX *vs*. control. OD, optic density. Protein loading was confirmed by the Ponceau S staining (Fig. S4).

In the 1W-DOX group, no changes in p38 MAPK, COX-2, and myeloperoxidase expression compared to 1W-Control mice were seen (Fig. 8a-c). In the 5M-DOX group, a significant increase in myeloperoxidase expression (Fig. 8f), a significant decrease in COX-2 expression (Fig. 8e), and a tendency for decreased (*p* = 0.088) p38 MAPK expression (Fig. 8d) were seen in comparison with the 5M-Control mice.

### The 1W-DOX Group Showed a Significant Increase in the NF-ĸB p65 Subunit

**Fig. 9:**
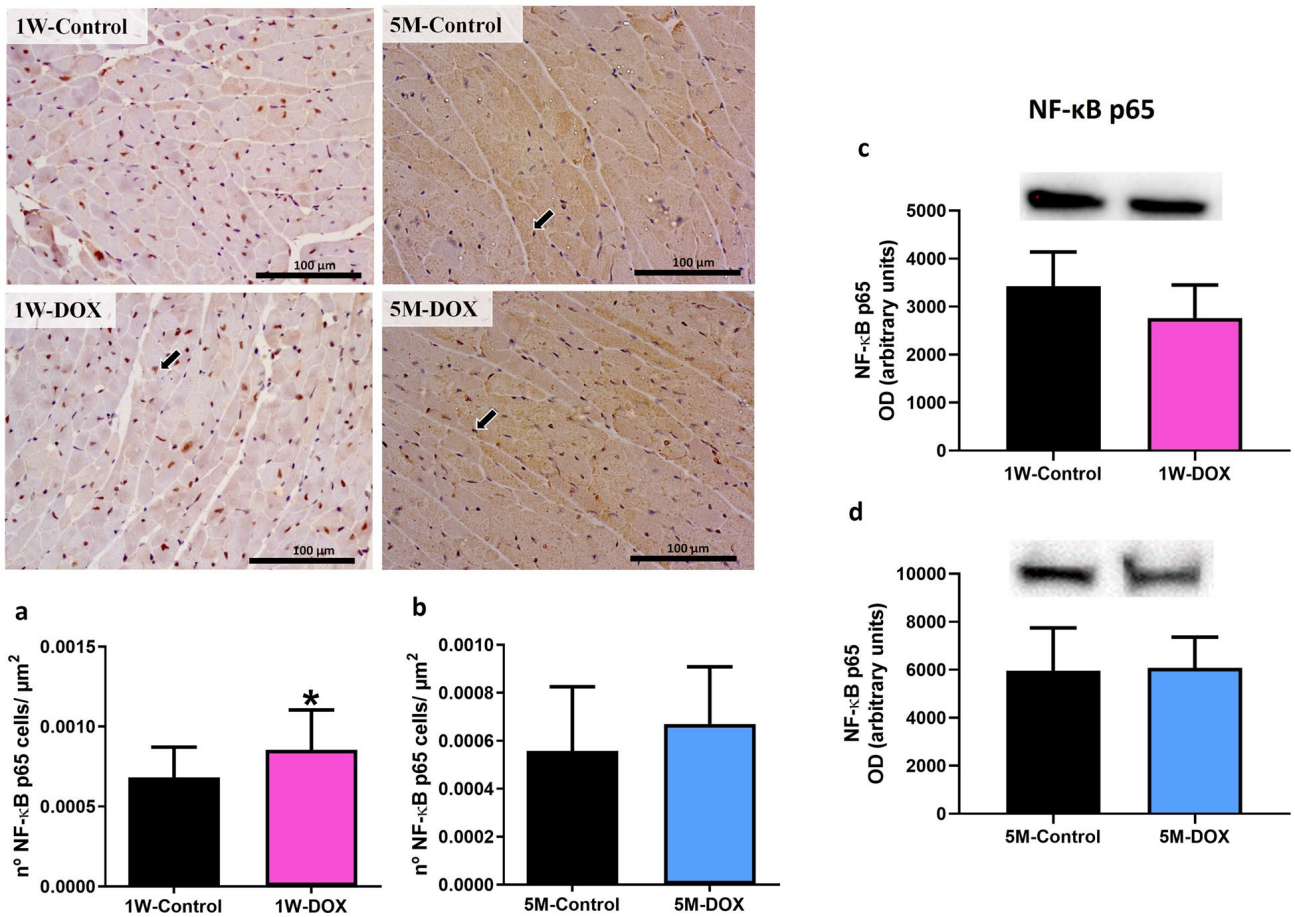
Representative images of the immunohistochemistry determination of nuclear factor κB (NF-κB) in the cardiomyocytes-like cells from mice sacrificed 1 week after the last administration of DOX (1W-DOX) and control mice (1W-Control), and mice sacrificed 5 months after the last administration of DOX (5M-DOX) and respective controls (5M-Control). **a**, **c** The number of cells staining as positive, indicated by black arrows for the activated NF-κB of the heart of DOX-treated and control groups, **a** 1W-DOX and 1W-Control; and **c** 5M-DOX and respective 5M-Control. Scale bar = 100 µm, *n* = 3 animals *per* group, six random fields *per* animal. Images were taken at 40 × magnification. The results were expressed according to the number of positive cells *per* area (µm^2^) as mean ± SD. Statistical comparisons were made using the Mann–Whitney test: *p < 0.05, DOX *vs*. control. **b**, **d** Western blotting analysis of NF-κB p65 (60 kDa) expression in the cardiac tissue, in **b** 1W-DOX and 1W-Control; and **d** 5M-DOX and 5M-Control. Values are expressed as mean ± SD and were obtained from 6 (1W) to 7 (5M) animals from each treatment group. Statistical comparisons were made using the unpaired *t*-test. OD, optic density. Protein loading on the Western blot was confirmed by the Ponceau S staining (Fig. S5).

**Fig. 10:**
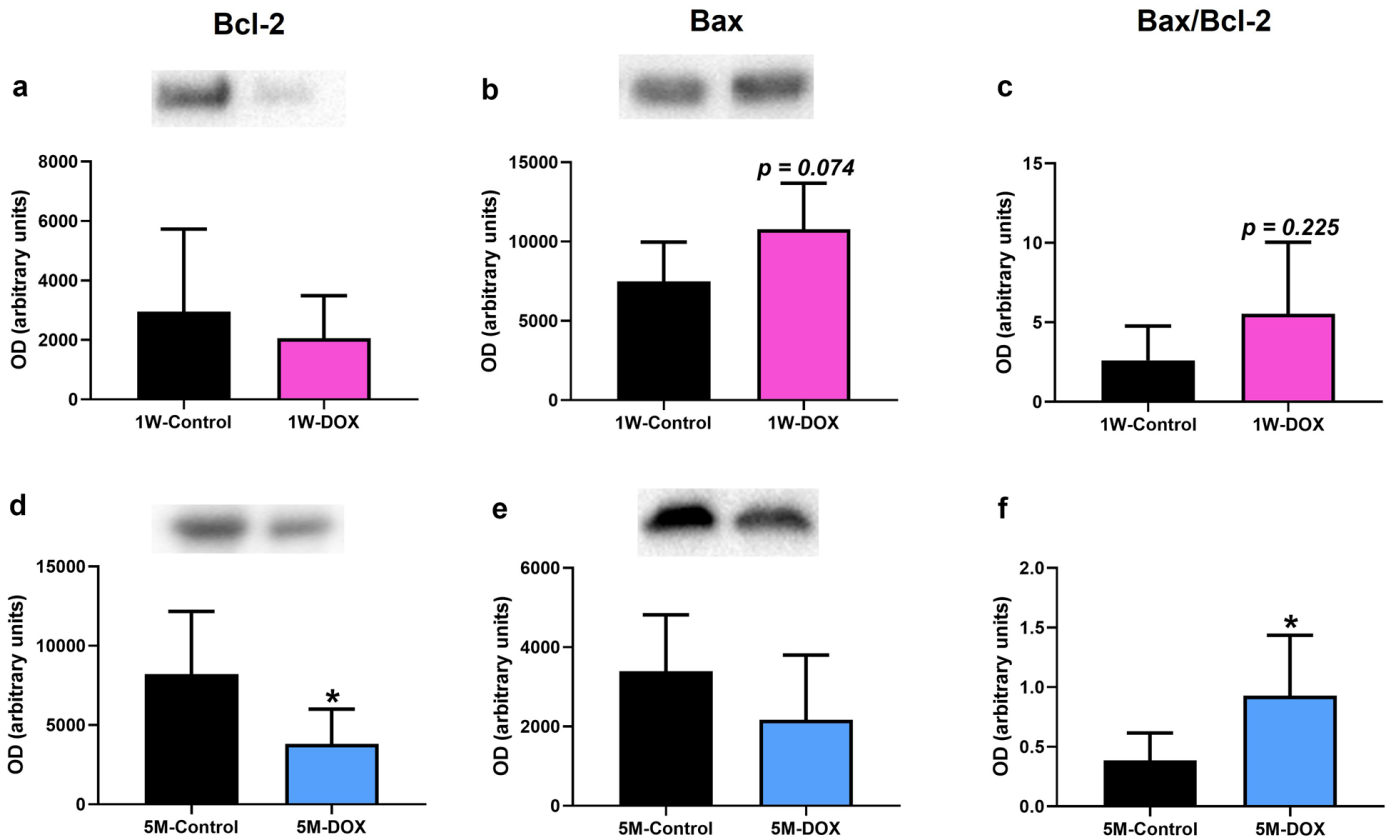
Western blotting analysis of **a**, **d** B-cell lymphoma 2 (Bcl-2) (26 kDa) and **b**, **e** B-cell lymphoma 2 associated X (Bax) (21 kDa) and **c**, **f** ratio of Bax/Bcl-2 expression in the cardiac tissue, in **a**, **b**, **c** mice sacrificed 1 week after the last administration of DOX (1W-DOX) and control mice (1W-Control); and **d**, **e**, **f** mice sacrificed 5 months after the last administration of DOX (5M-DOX) and respective controls (5M-Control). Values are expressed as mean ± SD and were obtained from 4–6 (1W) to 4–7 (5M) animals from each treatment group. Statistical comparisons were made using the unpaired *t*-test: *p < 0.05, DOX *vs*. control. OD, optic density. Protein loading was confirmed by the Ponceau S staining (Fig. S6).

In the 1W-DOX group, a significant increase in the number of NF-κB p65 cells was observed when compared with the respective 1W-Control mice (Fig. 9a). The 1W-DOX group exhibited positive cytoplasmic and nuclear expressions (brown staining) when compared with the 1W-Control mice (Fig. 9a). These results were supported by semi-quantitative analysis, where the 1W-DOX mice (Fig. 10a) had a higher amount of NF-κB immunopositive cells (*p* < 0.05) when compared with the respective control group. In the 5M-DOX group, no meaningful differences were seen (Fig. 9b). When total homogenates of cardiac tissue were analyzed via Western blot, no differences were observed in NF-κB p65 expression in either group (Fig. 9c, d).

### The 5M-DOX Group Showed a Significant Decrease in Bcl-2 Expression

The 1W-DOX group showed no meaningful differences in B-cell lymphoma 2 (Bcl-2) expression, compared to 1W-Control mice (Fig. 10a) although a tendency for increased (*p* = 0.074) B-cell lymphoma 2 associated X (Bax) expression was seen, compared to 1W-Control mice (Fig. 10b). In the 5M-DOX group, a significant decrease in Bcl-2 expression in comparison with the control group (Fig. 10d) was observed, while no changes in Bax expression (Fig. 10e) were seen. The ratio of Bax/Bcl-2 (pro-apoptotic parameter) increased significantly in 5M-DOX mice when compared with the 5M-Control mice (Fig. 10f).

## Discussion

This work is the first to make a study of the impact of DOX in CD-1 male mice 5 months after the last administration. The major findings of this work were (1) even 5 months after DOX treatment, the body weight was still affected in mice; (2) histopathological examination allowed to see cardiac injury at 1W after DOX; (3) both DOX-treated groups had a significant increase in fibrotic tissue compared to control; (4) glutathione peroxidase, catalase, and iNOS expression increased in the 1W-DOX group, while in the 5M-DOX group, an increase in SOD2 expression and a tendency for the increase of catalase expression were seen; (5) the 1W-DOX group, a significant decrease in Nrf2 expression and a significant increase in p62 expression were observed, while in the 5M-DOX group, a significant increase in Nrf2 expression was seen; (6) in the 1W-DOX group, a tendency for an increase in Bax expression was seen, while in the 5M-DOX group, a significant decrease in Bcl-2 expression and an increase in the ratio of Bax/Bcl-2 expression were found. Regarding markers of inflammation: (1) in both DOX-treated groups, a higher density of infiltrating M1 macrophages was seen, although only the 1W-DOX group had a higher number of NF-κB p65 immunopositive cells in the cardiac tissue; (2) in the 1W-DOX group, a significant increase in TNFR2 expression and a tendency for increased TNFR1 and IL-6 expression were observed; (3) in the 5M-DOX group, a significant decrease of heart COX-2 expression, a tendency for decreased p38 MAPK expression, and a significant increase of myeloperoxidase, IL-33, and TNF-α expression were detected.

While in the 1W-DOX group, the body weight was constant throughout the experiment after 9.0 mg/kg of DOX, in the animals 5M-DOX, there was a significant body weight decrease in the last days of the protocol. To the best of our knowledge, no long-term study like this was previously done and we demonstrated that although a low cumulative dose (corresponding to 54.45 mg/m^2^ in humans) has been used, DOX has a strong impact months after administration.

In the present work, we observed that even at a 9 mg/kg cumulative dose of DOX, cardiac tissue lesions were seen. Similar results were reported in our previous work [19] and by other authors [37–45], when higher cumulative doses were used, revealing cellular damage, mostly swollen and vacuolated cardiomyocytes, capillary congestion, interstitial oedema, disorganization of myofibrils, and interstitial inflammatory cell infiltration, in different degrees. On the other hand, a semi-quantitative histopathologic analysis of the heart showed that it seems the heart had partially recovered, at least when assessing the numerical values of the semi-quantitative analysis from DOX treatment at 5M. Nevertheless, we observed that DOX treatment induced a significant increase in collagen deposition in the cardiac tissue of mice sacrificed 1W and 5M after the last administration. This result is according to what has been previously published in animal models [19, 46, 47].

Oxidative stress is the most frequently proposed mechanism to explain the complex pathophysiology of DOX-induced cardiotoxicity [2]. The heart is very prone to oxidative damage due to low levels of antioxidant enzymes. Although some studies have shown that the treatment of animals with antioxidants protects the heart against the toxicity of DOX [48, 49], no antioxidants have been proven to have clinical efficacy [50]. Conversely, the literature has descriptions that overexpression of antioxidant enzymes such as SOD2, catalase, or glutathione peroxidase in cardiomyocytes of transgenic mice attenuates DOX-induced cardiac damage [51–53]. In the present work, DOX did not alter SOD2 expression but significantly increased catalase and glutathione peroxidase expression in the heart of the 1W-DOX group. Interestingly, DOX-induced contractile and mitochondrial dysfunction in the heart of mice were prevented by the overexpression of glutathione peroxidase [53] and what we see in our present work may be an adaptative response to cope with DOX-inflicted cardiotoxicity. In the 5M-DOX group, only an increase in SOD2 expression and a tendency to increase catalase expression were observed. Increased SOD2 expression has been shown to protect mitochondria from oxidative damage, decrease apoptosis, and preserve left ventricular function [54, 55]. Overall, our results suggest that DOX induces different responses regarding redox homeostasis. Nonetheless, we observed that protein oxidation by carbonylation was not seen in the 1W-DOX group, possibly because of the lower cumulative dose, when compared to previous studies [19, 37, 38, 56, 57], in which increased cardiac protein carbonylation was seen. Conversely, the 5M-DOX group had decreased carbonylated protein expression, showing that repair responses were triggered as seen in our previous study [19]. Possible, activation of the proteasome system acting on oxidatively modified proteins or other mechanisms may be involved that still need to be clarified.

iNOS is expressed in the heart upon inflammatory stimuli to engage in the production of excessive amounts of ^•^NO [58] and it is considered to be a marker for oxidative/nitrosative stress [59]. In studies with mice that received DOX (20 mg/kg ip), a marked reduction in cardiac contractility was observed 5 days after administration, associated with a significant increase in myocardial iNOS immunopositivity and 3-nitrotyrosine formation [60, 61]. In addition, Mukhopadhyay and co-workers observed an increase in myocardial iNOS expression after DOX (20 mg/kg body weight ip) administration that peaked around day 5 when myocardial dysfunction was evident [62]. Moreover, the literature shows that iNOS induction plays a pathogenetic role in the development of chronic DOX-induced HF [63]. However, other studies have shown that iNOS-derived ^•^NO may have protective effects on the heart, such as reducing myocardial damage after ischemia–reperfusion injury [64]. Although contradictory data may arise from other works, herein iNOS expression increased in the mice heart after DOX (1W-DOX group), and after the initial increase of iNOS expression, a normalization of response was seen, and no meaningful changes were observed in iNOS expression in the 5M-DOX group as compared with controls. Overall, the role of iNOS in the heart is complex and context-dependent, and we cannot take robust conclusions on its role on the cardiotoxicity seen here.

While oxidative stress has been linked to DOX cardiotoxicity, one of the key players of redox homeostasis is Nrf2 [63]. ROS generation is counteracted by cytoprotective mechanisms via the regulation of KEAP1/Nrf2 signaling [65]. Under physiological conditions, and through the canonical KEAP1/Nrf2 pathway, Nrf2 is suppressed by the negative regulator KEAP1, which leads to its ubiquitylation and proteasomal degradation. On the other hand, when Nrf2 escapes from the KEAP1 interaction, it translocates into the nucleus, where it regulates the expression of antioxidant and anti-inflammatory genes [66]. Nonetheless, several non-canonical pathways for Nrf2 activation engage competitive inhibition of the KEAP1/Nrf2 interaction by intracellular proteins such as p62 [67]. p62 is involved in various cellular processes, including autophagy, and signaling pathways related to inflammation and oxidative stress [68]. p62 sequesters KEAP1 within autophagosomes, preventing ubiquitylation of Nrf2 and leading to the release of Nrf2 into the nucleus. Therefore, the p62-KEAP1-Nrf2 signaling pathway plays an important role in the cellular response to oxidative stress [69, 70]. In this work, in the 1W-DOX group, a significant increase in p62 expression was observed; however, Nrf2 is contra-intuitively decreased. Decreased Nrf2 expression could potentially limit its ability to counteract oxidative stress and inflammation [71]. Herein, p62 does not seem to directly affect Nrf2, being possibly involved in other pathways *e.g.* autophagy. A study by Li and collaborators showed that Nrf2 deficiency exacerbates DOX-induced cardiotoxicity and cardiac dysfunction, suggesting that Nrf2 could be an endogenous suppressor of DOX-induced cardiotoxicity by controlling both oxidative stress and autophagy in the heart [24]. On the other hand, in the present work in the 5M-DOX group, a significant increase in Nrf2 expression was seen, corroborating a fully active proteasome in this time course. Moreover, while Bax was increased in the short term, its levels have normalized in the long term, indicating a potential shift in the regulation of cell survival and apoptosis. Although other mechanisms can be involved, in the 5M-DOX group (long-term), the activation of Nrf2 seems to be cardioprotective and interacts with apoptotic genes and possibly contributing to the damage recovery. 

Nrf2 and NF-κB are key pathways regulating the balance of cellular redox status and responses to stress and inflammation [72]. NF-κB activity is limited when Nrf2 activation occurs [28]. In contrast, the inactivation of Nrf2 leads to the loss of NF-κB suppression and therefore upregulation of inflammatory responses [28]. In the 1W-DOX group, a higher density of infiltrating M1 macrophages and a higher number of NF-κB p65 immunopositive cells were seen with lower Nrf2 expression. M1 macrophages are required for the induction of a large number of inflammatory genes, including TNF-α, IL-1β, IL-6, IL-12p40, and COX-2, involved in various inflammatory processes [73], which may result in cardiac damage. The literature shows that the levels of pro-inflammatory cytokines, IL-1β [20, 41, 74], IL-6 [74], and TNF-α [41, 74] are increased after DOX administration (mostly after higher cumulative doses than ours study), and those are implicated in cardiac pathogenesis and apoptosis. The 1W-DOX mice showed a significant increase in TNFR2 expression and a tendency for an increase in TNFR1 and IL-6 expression. TNFR1 promotes biological responses ranging from NF-κB activation to cell death [75, 76], corroborating our data of NF-κB and Bax described earlier. In our previous work, DOX-treated adult mice (18.0 mg/kg cumulative dose) showed a tendency towards increased TNFR2, perhaps because this receptor activates NF-κB [19]. On the other hand, the heart was able to cope with the initial inflammatory stimuli caused by DOX, seen by increased expression of TNFR2, and a tendency for increased TNFR1 and IL-6 expression. However, in the 5M-DOX group, no meaningful differences in NF-κB p65, IL-1β, IL-6, TNFR1, and TNFR2 expression were seen. In fact, although apparently contra-intuitive in the 5M-DOX group, an increase in M1 macrophages was seen, while no changes in NF-κB p65 immunopositive were detected, and simultaneously Nrf2 increased expression. Thus, we hypothesize that DOX may have triggered, even at this low cumulative dose, inflammatory mediators, and some persisted throughout 5 months but were mainly abrogated (with the exception of M1 cells and related myeloperoxidase, TNF-α and IL-33). IL-33 is a cytokine produced by cardiac fibroblasts that can cause cardioprotective effects against hypertrophic remodeling and myocardial fibrosis by antagonizing angiotensin-II signaling and promoting anti-apoptotic factors, respectively [77, 78]. Moreover, in a model of recurrent neonatal seizure, IL-33 provided protection by suppressing apoptosis, and NF-κB-mediated inflammatory pathways, while maintaining p62 in normal levels [79].

Recent studies have shown that COX-2 and Nrf2 may interact to regulate inflammation and oxidative stress in the heart. COX-2 is an enzyme that is released at the site of tissue injury to produce prostaglandin E2 that stimulates inflammation. COX-2-derived prostaglandins have been shown to inhibit Nrf2 activity, leading to increased oxidative stress and inflammation in the heart [80, 81]. In contrast, Nrf2 activation in more severe and prolonged states of oxidative stress has been shown to inhibit COX-2 expression and inflammatory mediators [82], leading to reduced inflammation and oxidative stress. In fact, at 5M we see that an increase in Nrf2 expression is accompanied by COX-2 levels decrease. In the present work, no changes were seen in COX-2 expression in the heart at the earlier time point, but it decreased in DOX animals after 5M. Saito *et al*. have suggested that the inhibition of COX-2 with a selective COX-2 inhibitor improves cardiac function when given after the period of acute infarction in some experimental rodent models [83]. Another study showed that a selective COX-2 inhibitor given before and during the period of acute infarction reduced macrophage infiltration and fibroblast proliferation in rats’ hearts [84]. Scheuren *et al.* [84] suggested that angiotensin-II regulates COX-2 expression through the p38 MAPK pathway in isolated cardiac fibroblasts. We observed that DOX-treated mice (5M-DOX) had a tendency towards decreased p38 MAPK expression, which may be correlated with the decreased expression of COX-2. Overall, the results here presented suggest that the decrease of COX-2 at DOX-5M could help mitigate injury and inflammatory responses, and it is suggestive of adaptation of the heart to DOX-induced cardiotoxicity over time. The results of this study represent new insights into the effects of DOX-induced cardiotoxicity at different time points that could help healthcare professionals address patient care. It also is important for healthcare providers to consider several factors, such as hypertension, diabetes, coronary artery disease, HF, and smoking, as risk factors for DOX-induced cardiotoxicity as well as concomitant pharmacotherapy [1] when treating patients with DOX. Only the full knowledge of the patients will allow them to take the right steps to minimize the risk of cardiotoxicity. Several modulators of the inflammatory response such as non-steroidal anti-inflammatory drugs, glucocorticoids, natural products [85], omega-3 fatty acids, and probiotics [86] can modify the effects of DOX treatment and affect the prognosis.

## Conclusion

Our study demonstrated that, in the short-term evaluation, DOX induces pathways that generate adverse outcomes related to inflammatory processes and response to oxidative stress. However, months after the last administration, the heart activates other response mechanisms. Nonetheless, some inflammatory mediators continued over time, and we cannot ignore possible chronic inflammation. In the heart of the 1W-DOX group, a significant decrease in Nrf2 expression was observed, a significant increase in p62 expression, and a tendency for an increase of Bax, suggesting that the apoptotic route is initiated. A higher number of NF-κB p65 immunopositive cells may suggest an interaction between these factors in the cellular response to oxidative stress and inflammation, with Nrf2 blockage and activation of the apoptosis pathway. On the other hand, in the 5M-DOX group, a significant increase in Nrf2 expression was found, and normalization of Bax expression, although M1 cells are still present and possibly are still removing debris of apoptotic cells of the earlier damage. These results suggest that in the 5M-DOX group, the activation of Nrf2 seems to be cardioprotective. Moreover, in the 5M-DOX group, DOX led to a reduction in COX-2 expression and an increase in IL-33 expression, which may contribute to mitigating fibrosis and inflammatory responses. This DOX cumulative dose may have led to an imbalance on redox defenses earlier on, possibly related to inflammation, which activates Nrf2. However, it is important to note that changes in Nrf2 expression, p62 expression, and related factors are likely reactive responses of the organism to the initial damage induced by DOX. Furthermore, we should consider the possibility that inflammation may result from the initial damage caused by cardiotoxicity, rather than being the primary instigator of cardiotoxicity itself. We believe that the present study provides valuable information on the potential mechanisms underlying DOX-induced cardiotoxicity; however, we recognize several limitations to the work that deserve consideration, namely the assessment of cardiotoxicity in this study is mainly based on semi-quantitative histopathology and no functional assessment of heart function was done. Moreover, although we hypothesize that inflammation and the Nrf2 modulation can be key to the cardiotoxicity and pathways changes observed, one cannot overrule other molecular pathways not evaluated here as important also for the crosstalk that leads to DOX-induced cardiotoxicity.

### Supplementary Information

Below is the link to the electronic supplementary material.

Supplementary file1 (DOC 41388 KB)

## Data Availability

The datasets generated or analyzed during the current study are available on request from the corresponding authors.
