# Supplementary material for: The Role of Nrf2 and Inflammation on the Dissimilar Cardiotoxicity of Doxorubicin in Two-Time Points: a Cardio-Oncology In Vivo Study Through Time
Source: Inflammation. 2023 Oct 14;47(1):264–84. doi: 10.1007/s10753-023-01908-0 (PMC10799157; doi:10.1007/s10753-023-01908-0)
Supplement: Supplementary file 1 — Supplementary file1 (DOC 41388 KB) [file 10753_2023_1908_MOESM1_ESM.doc]

Supplementary Materials

***The role of Nrf2 and inflammation on the dissimilar cardiotoxicity of doxorubicin in two-time points: a car******dio-oncology in vivo study through time***

Ana Reis-Mendes^a,b,*^, Mariana Ferreira^a,b^,, Ana Isabel Padrão^c^, José Alberto Duarte^c,d^, Margarida Duarte-Araújo^e,f^, Fernando Remião^a,b^, Félix Carvalho^a,b^, Emília Sousa^g,h^, Maria Lourdes Bastos^a,b^ and Vera Marisa Costa^a,b,*^

^a^Associate Laboratory i4HB - Institute for Health and Bioeconomy, Laboratory of Toxicology, Department of Biological Sciences, Faculty of Pharmacy, University of Porto, Porto, Portugal; ^b^UCIBIO - Applied Molecular Biosciences Unit, REQUIMTE, Laboratory of Toxicology, Department of Biological Sciences, Faculty of Pharmacy, University of Porto, Porto, Portugal; ^c^Research Center in Physical Activity, Health and Leisure (CIAFEL), Laboratory for Integrative and Translational Research in Population Health (ITR), Faculty of Sport, University of Porto, Porto, Portugal; ^d^TOXRUN – Toxicology Research Unit, University Institute of Health Sciences, CESPU, CRL, Gandra, Portugal; ^e^LAQV/REQUIMTE, University of Porto, Porto, Portugal; ^f^Department of Immuno-Physiology and Pharmacology, Institute of Biomedical Sciences Abel Salazar, University of Porto, Porto, Portugal; ^g^Laboratory of Organic and Pharmaceutical Chemistry, Chemistry Department, Faculty of Pharmacy, University of Porto, Porto, Portugal; ^h^CIIMAR – Interdisciplinary Centre of Marine and Environmental Research, Porto, Portugal.

**Table S1.** Summary of the characteristics of the primary antibody used in the presented experiments.

| **Primary antibody** | **Manufacturer** | **Reference** | **Host Animal** | **Dilution for Immunohistochemistry** | **Dilution for Western Blot** |
| --- | --- | --- | --- | --- | --- |
| **B-cell lymphoma 2 associated x (Bax)** | Abcam (Cambridge, UK) | ab32503 | Rabbit, monoclonal | - | 1:1000 |
| **B-cell lymphoma 2 (Bcl-2)** | Santa Cruz Biotechnology (Heidelberg, Germany). | sc-7382 | Mouse, monoclonal | - | 1: 200 |
| **Catalase** | Abcam (Cambridge, UK) | ab16731 | Rabbit, polyclonal | - | 1:500 |
| **Cluster of differentiation 68 (CD68) (M1 macrophage)** | Abcam (Cambridge, UK) | ab125212 | Rabbit, polyclonal | 1:50 | - |
| **Cyclooxygenase-2 (COX-2)** | Abcam (Cambridge, UK) | ab15191 | Rabbit, polyclonal | - | 1:1000 |
| **Dinitrophenyl-KLH** | Invitrogen/Life Technologies (Grand Island, NY, USA) | A6430 | Rabbit, polyclonal | - | 1:1000 |
| **Glutathione peroxidase 1** | Abcam (Cambridge, UK) | ab22604 | Rabbit, polyclonal | - | 1:1000 |
| **Inducible nitric oxide synthase (iNOS)** | Abcam (Cambridge, UK) | ab15323 | Rabbit, polyclonal | - | 1:200 |
| **interleukin (IL)-1β** | Thermo Fisher Scientific (Waltham, MA, USA); | P420B | Rabbit, polyclonal | - | 1:1000 |
| **IL-33** | Abcam (Cambridge, UK) | ab187060 | Rabbit, monoclonal | - | 1:1000 |
| **IL-6** | Abcam (Cambridge, UK) | ab208113 | Rabbit, polyclonal | - | 1:1000 |
| **Mannose receptor (M2 macrophage)** | Abcam (Cambridge, UK) | ab64693 | Rabbit, polyclonal | 1:50 | - |
| **Myeloperoxidase** | Abcam (Cambridge, UK) | ab139748 | Rabbit, polyclonal | - | 1:500 |
| **Nuclear factor κB (NF-κB) p65** | Abcam (Cambridge, UK) | ab16502 | Rabbit, polyclonal | 1:50 | 1:1000 |
| **Transcription factor nuclear factor erythroid 2-related factor 2 (Nrf2)** | Abcam (Cambridge, UK) | ab31163 | Rabbit, polyclonal | - | 1:1000 |
| **p38 mitogen-activated protein kinase (MAPK)** | Cell Signaling Technology through Werfen (Carnaxide, Portugal) | #9212 | Rabbit, polyclonal | - | 1:1000 |
| **p53** | Santa Cruz Biotechnology (Heidelberg, Germany) | sc-6243 | Rabbit, polyclonal | - | 1:1000 |
| **p62** | Abcam (Cambridge, UK) | ab56416 | Mouse, monoclonal | - | 1:1000 |
| **Superoxide dismutase 2**  **(SOD2)** | Abcam (Cambridge, UK) | ab13534 | Rabbit, polyclonal | - | 1:500 |
| **Tumour necrosis factor-α (TNF-α)** | Abcam (Cambridge, UK) | ab66579 | Rabbit, polyclonal | - | 1:500 |

**Figure S1.** Western blotting analysis and loading control of Ponceau S staining of (A, E) Glutathione peroxidase (22 kDa), (B, F) catalase (60 kDa), (C, G) superoxide dismutase 2 (SOD2) (26.6 kDa) and (D, H) inducible nitric oxide synthase (iNOS) (131 kDa) from mice treated with DOX (D) or respective controls (C) sacrificed (A, B, C, D) 1 week or (E, F, G, H) 5 months after the last administration. The bands identified within the square correspond to the Western blot bands used as an example above the graphs in the original article.

**Figure S2.** Western blotting analysis and loading control of Ponceau S staining of (A, C) Nuclear factor erythroid-2 related factor 2 (Nrf2) (97 kDa) and (C, D) p62 (62 kDa) from mice treated with DOX (D) or respective controls (C) sacrificed (A, B) 1 week or (C, D) 5 months after the last administration. The bands identified within the square correspond to the Western blot bands used as an example above the graphs in the original article.

**Figure S3.** Western blotting analysis and loading control of Ponceau S staining of (A, E) interleukin-1 beta (IL-1 beta) (35 kDa), (B, F) Interleukin-6 (IL-6) (23 kDa), (C, G) Interleukin-33 (IL-33) (33 kDa), (D, G) Tumor necrosis factor- α (TNF-α) (25 kDa), (C, H) type 1 TNF receptor (TNFR1) (50 kDa) and (D, G) type 2 TNF receptor (TNFR2) (75 kDa) from mice treated with DOX (D) or respective controls (C) sacrificed (A, B, C, D) 1 week or (E, F, G) 5 months after the last administration. The bands identified within the square correspond to the Western blot bands used as an example above the graphs in the original article.

**Figure S4.** Western blotting analysis and loading control of Ponceau S staining of (A, D) p38 MAP Kinase (p38 MAPK) (40kDa), (B, E) cyclooxygenase 2 (COX2) (75 kDa), (C, F) Myeloperoxidase (63 kDa) from mice treated with DOX (D) or respective controls (C) sacrificed (A, B, C) 1 week or (D, E, F) 5 months after the last administration. The bands identified within the square correspond to the Western blot bands used as an example above the graphs in the original article.

**Figure S5.** Western blotting analysis and loading control of Ponceau S staining of (B, D) NF-κB p65 (60 kDa) from mice treated with DOX (D) or respective controls (C) sacrificed 1 week or 5 months after the last administration. The bands identified within the square correspond to the Western blot bands used as an example above the graphs in the original article.

**Figure S6.** Western blotting analysis and loading control of Ponceau S staining of (A, C) B-cell lymphoma 2 (Bcl-2) (26 kDa) and (B, D) B-cell lymphoma 2 associated x (Bax) (21 kDa) from mice treated with DOX (D) or respective controls (C) sacrificed (A, B) 1 week or (C, D) 5 months after the last administration. The bands identified within the square correspond to the Western blot bands used as an example above the graphs in the original article.
